# Pectin Hydrogels: Gel-Forming Behaviors, Mechanisms, and Food Applications

**DOI:** 10.3390/gels9090732

**Published:** 2023-09-09

**Authors:** Nurul Saadah Said, Ibukunoluwa Fola Olawuyi, Won Young Lee

**Affiliations:** 1 School of Food Science and Technology, Kyungpook National University, Daegu 41566, Republic of Korea; nurulsaadah.said@gmail.com (N.S.S.); ifolawuyi@knu.ac.kr (I.F.O.); 2 Research Institute of Tailored Food Technology, Kyungpook National University, Daegu 41566, Republic of Korea

**Keywords:** pectin, gels, gelling mechanism, hydrogel, application

## Abstract

Pectin hydrogels have garnered significant attention in the food industry due to their remarkable versatility and promising properties. As a naturally occurring polysaccharide, pectin forms three-dimensional (3D) hydrophilic polymer networks, endowing these hydrogels with softness, flexibility, and biocompatibility. Their exceptional attributes surpass those of other biopolymer gels, exhibiting rapid gelation, higher melting points, and efficient carrier capabilities for flavoring and fat barriers. This review provides an overview of the current state of pectin gelling mechanisms and the classification of hydrogels, as well as their crosslinking types, as investigated through diverse research endeavors worldwide. The preparation of pectin hydrogels is categorized into specific gel types, including hydrogels, cryogels, aerogels, xerogels, and oleogels. Each preparation process is thoroughly discussed, shedding light on how it impacts the properties of pectin gels. Furthermore, the review delves into the various crosslinking methods used to form hydrogels, with a focus on physical, chemical, and interpenetrating polymer network (IPN) approaches. Understanding these crosslinking mechanisms is crucial to harnessing the full potential of pectin hydrogels for food-related applications. The review aims to provide valuable insights into the diverse applications of pectin hydrogels in the food industry, motivating further exploration to cater to consumer demands and advance food technology. By exploiting the unique properties of pectin hydrogels, food formulations can be enhanced with encapsulated bioactive substances, improved stability, and controlled release. Additionally, the exploration of different crosslinking methods expands the horizons of potential applications.

## 1. Introduction

In recent times, there has been a growing interest in developing innovative biomaterials derived from natural biopolymers that could potentially revolutionize the food sector by improving product quality and providing functional advantages to customers. Among these biomaterials, carbohydrate-based polymers such as starch, cellulose, chitosan, and pectin have emerged as versatile and valuable assets in food technology [1,2,3,4,5]. These naturally derived polymers offer a wide range of applications as stabilizers, fat replacers, emulsifiers, etc., in the food science industry, and they contribute distinct functionalities to food products. In addition, they are applicable for ingredient enhancement, additive integration, and food packaging [6,7]. For instance, starch and cellulose can contribute as dietary fiber, act as an effective thickening and stabilizing agent [8,9], or be employed as materials for food packaging [10,11]. Chitosan, with its potent antimicrobial properties, is used as a preservative packaging material for extending the shelf life of perishable goods, particularly fruits and vegetables [12]. On the other hand, pectin, renowned for its remarkable gelling properties, frequently serves as a crucial gelling agent, transforming liquid formulations into stable gels, imparting desirable textures, and enhancing product quality [13]. Besides these diverse applications, the preparation of food hydrogels using these biopolymers has recently garnered significant interest in the food industry.

Hydrogels are polymeric materials with a 3D network capable of absorbing and retaining water, making them highly hydrophilic [14]. Besides their controlled release properties, ability to provide structural/textural stability, and ability to mimic desired food textures, they are indispensable in modern food technology, further extending their applications to other fields, including cosmetics, drug delivery, and tissue engineering [15,16,17]. Hydrogels are employed for tasks such as enabling intricate shapes in 3D printing, serving as fat substitutes, and promoting satiety with smaller portions [18,19]. They also serve as thickeners and stabilizers due to their capacity to retain a substantial quantity of water or polar solvents while maintaining a solid-like structure, achieved through the physical or chemical crosslinking of hydrophilic polymer chains. 

Pectin, a naturally occurring polysaccharide found in plant cell walls, is primarily composed of repeating units of α-(1-4)-linked D-galacturonic acid units [20]. Depending on the plant source, a pectin structure may consist of homogalacturonan (HG), rhamnogalacturonan I (RG-I), and rhamnogalacturonan II (RG-II) domains [21]. This diversity in composition accounts for the unique properties exhibited by different pectins, such as their gelation, solubility, and rheological behavior. Additionally, pectin can form 3D networks of hydrophilic polymer chains, making it ideal for preparing hydrogels. Gels prepared using pectin are advantageous over other biopolymer gels, such as gelatin, in terms of their ability to form gels rapidly, elevated thermal stability, and exceptional capacity for encapsulating flavors and creating fat barriers [22]. Compared to other natural biopolymers such as starch, cellulose, chitosan, collagen, protein, and agarose, pectin distinguishes itself through its exceptional gelling properties, allowing for the creation of stable hydrogels under milder conditions. Moreover, pectin provides the advantage of controllable gelation and interactions through its adjustability by modifying its degree of methoxylation and acetylation [23,24,25]. Also, its amphiphilic nature, with both polar and non-polar sites within its structure [26], enables effective interaction with water and oil, making it versatile for encapsulating hydrophobic bioactives. 

In application, pectin hydrogels are currently being investigated for their potential in the development of innovative food products and the fabrication of structured food with specific textures for specific purposes while elevating sensory properties. Also, there is evidence that pectin hydrogels can be employed for effective encapsulation and targeted release of bioactives to specific digestive tract regions [27]. Therefore, this paper aims to provide valuable insights through a systematic review of the present status of research and advancements in pectin hydrogels. This review could stimulate further exploration and utilization of pectin hydrogels, aligning with the evolving demands of modern consumers and promoting advancements in food technology.

## 2. Pectin Extraction and Characterization

### 2.1. Extraction of Pectin from Various Sources

Pectin can be extracted from a diverse range of sources, each possessing its own distinctive composition and properties. Among these sources, pectin is commonly obtained from citrus fruits like oranges, lemons, and limes due to their substantial peel content and higher yield of pectic polysaccharides (Table 1). Also, apples offer a pectin-rich pomace that includes both peels and cores, contributing to their relatively high pectin yield. Other alternative sources, such as bananas, potato pulp, pumpkin peels, watermelon rinds, cocoa husks, and soy hulls, have also been investigated for their pectin content. These unconventional sources hold promise for diversifying pectin extraction options and utilizing agricultural byproducts effectively.

Recently, pectin extraction studies have progressively focused on the upcycling of byproducts from the fruit industry. By valorizing the value of peel waste and husks, which are often discarded, these byproducts offer an eco-friendly and sustainable avenue for pectin extraction. This approach aligns with the principles of the circular economy, turning what was once considered waste into a valuable resource.

Pectin is typically extracted through aqueous methods (Table 1), including conventional heating (CE) [31,34], microwave heating (MAE) [29,30], ultrasonic (UAE) [37,43], and enzymatic extraction (EAE) methods [28]. Unconventional techniques like ultra-high pressure (UHP) and subcritical water (SW) extraction have also been explored, but they can lead to some degree of pectin quality limitation and degradation [31,56]. It is worth noting that pectin yield is also influenced by factors like temperature, extraction time, pH, and raw material characteristics, along with extraction parameters [57].

### 2.2. Pectin Structure and Characterization

Pectin, being a complex polysaccharide, has an extensive variety of physical and chemical configurations, which affect its properties and functionality in a diverse array of food applications. Pectin is mostly made of α-(1,4)-linked D-galacturonic acid units, which form the polysaccharide’s backbone [58]. Moreover, pectin molecules exhibit pendant groups that encompass both hydrophilic functional units, including hydroxyl and carboxyl groups, and hydrophobic functional units, such as carboxylic ester and amide groups [59]. The polysaccharide chain found in pectin is hydrophilic [60], while proteins, feruloylated groups, and methyl and acetyl groups in pectin molecules are hydrophobic, which gives pectin good amphiphilic properties [59,60,61]. Among the pendant groups in pectin’s backbone structure, acetyl groups (CH_3_CO–) and methoxy groups (CH_3_O–) are the most prevalent, as they play essential roles in defining the functionality of pectin. The acetyl groups are typically attached to the hydroxyl (-OH) groups of galacturonic acid units within the pectin structure. This is typically presented as the degree of acetylation (DA), which is the number of acetyl groups present per galacturonic acid unit [62]. The presence of acetyl groups profoundly influences pectin’s solubility, gelation characteristics, and interactions with other molecules. Higher acetylation levels can impede molecular interactions among pectin molecules, leading to a reduced ability to form gels [63,64]. Conversely, lower acetylation levels encourage stronger interactions, enhancing pectin’s gelling properties and potential applications. In contrast, methoxy groups are linked to the carbon atoms of galacturonic acid units, and the degree of methoxylation (DM) signifies the number of methoxy groups per galacturonic acid unit [65,66,67]. Methoxylation considerably affects the gelation behavior of pectin [68], especially in the presence of calcium ions, which will be further explained in subsequent sections that focus on the pectin gelling mechanism.

**Figure 1:**
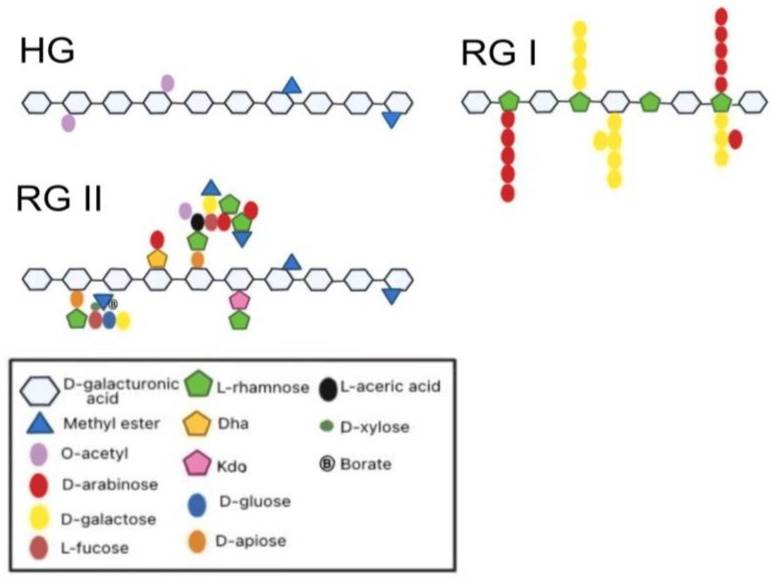
Schematic diagram of various pectin structures.

Based on the degree of esterification (DE) of these groups, pectin is classified either as low-methoxyl (LMP; DE 50%) or high-methoxyl (HMP; DE > 50%) [69]. The DE influences the gelation behavior, solubility, and engagement with other constituents within food matrices, making it a critical parameter in tailoring pectin for specific applications. The presence of side branches and neutral sugar side chains, such as rhamnose and arabinose residues, also has a substantial impact on pectin’s gelling property, influencing its overall structure and activity [70,71,72]. Pectin consists of three main regions (Figure 1): homogalacturonan (HG), which is regarded as the “smooth” region and made up of α-(1,4)-linked D-galacturonic acid residues with methyl and acetyl esterification; rhamnogalacturonan I (RG-I), which is termed the “hairy” region with alternating L-Rha and D-GalA residues and variable side chains; and rhamnogalacturonan II (RG-II), a complex structure containing up to 13 different sugars and 21 glycosidic linkages [73]. 

HG, a linear chain of α-(1,4)-linked galacturonic acid residues, is the primary structural component of pectin [74]. HG plays a pivotal role in establishing the gel matrix of pectin hydrogels. Commercial pectin, with a higher proportion of the HG domain, is renowned for its superior gelling ability, making it a widely used and stable gel-forming agent in the food and pharmaceutical industries [75]. The gelation mechanism includes divalent cations, such as calcium ions, interacting with the carboxyl groups present in the galacturonic acid residues. This results in the creation of “egg-box” structures in which calcium ions bridge adjacent HG chains, resulting in a 3D gel network with improved strength and stability. Also, for pectin consisting of both HG and RG-I structures, its ability to form gels is influenced by the ratio of HG:RG-I and the length of the HG domain. In addition, it has been reported that hydrogels exhibit optimal properties when prepared using pectin with a high content of galacturonic acid (GalA), a high molecular weight, and a suitable proportion of side chains (>15.8%) [76].

RG-I is a complex side chain composed of alternating galacturonic acid and rhamnose residues that is connected to the HG backbone [77]. The length of the RG-I backbone can range between 20 and 300 repeating units. At the C-4 position of rhamnose residues in pectin, there are side chains composed of galactose and/or arabinose residues. These side chains can form individual sugar units or combine to create chains of arabinans, galactans, or arabinogalactans. The branched rhamnose units in these chains account for 20–80% of the total structure [67,78]. A study by Zheng et al. [70] demonstrated that RG-I-enriched pectin may produce gels under both cation-induced and acid-induced circumstances. The existence of numerous arabinose sugar side chains in RG-I contributes significantly to the gel network’s strength by generating entanglements and stabilizing both chain–chain and dimer–dimer structures. These side-chain entanglements provide a denser gel network, limiting network chain mobility, and strengthening hydrophobic and hydrogen bonding in the HG region, which results in enhanced gel strength.

RG-II is the most complex and structurally unique component of pectin. It consists of a branched backbone of alternating galacturonic acid and rhamnose residues, with various side chains containing arabinan, apiose, and xylose residues [67,74,77]. RG-II has great crosslinking capabilities [79], which help to generate robust and stable pectin hydrogels. Because of its structural complexity, RG-II may interact with a wide spectrum of macromolecules in food systems, making it a crucial element in improving the functionality and performance of pectin hydrogels. However, there are yet few studies on hydrogels prepared with RG-II pectins or pectin structures containing a higher proportion of RG-II.

## 3. Gelling Mechanism of Pectin

The gelling properties and mechanisms of pectin have primarily been categorized and discussed in various studies, with a focus on factors such as its degree of esterification. This section discusses the impact of the presence of esters and other molecules in the pectin network on its gelation behavior and structural characteristics. 

### 3.1. High-Ester Pectin

**Figure 2:**
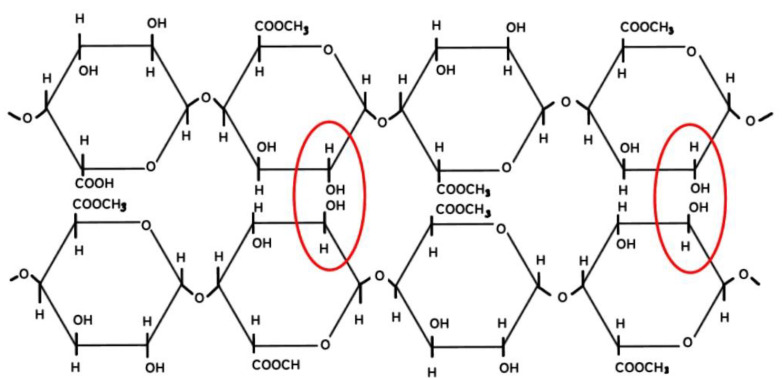
Gelling mechanism of high-methoxyl pectin. Red circle represents hydrogen bond formation between the pectin chains.

High-methoxyl (HM) pectins, with a DE ranging from 50% and above, predominantly form gels through the cohesion of hydrophobic forces and the formation of hydrogen bonds under specific environmental circumstances. These conditions include low pH levels (around 2.5 to 3.5) and the presence of soluble solids like sucrose (55% to 75%) or similar co-solutes for the gelling process to occur [74,80,81]. Sugar plays a crucial role in gel formation by reducing the amount of available water, which stabilizes the junction zones through hydrophobic interactions. As a result, the formed gel exhibits a two-dimensional (2D) network of interconnected pectin molecules with water and co-solutes trapped inside, contributing to its ability to resist deformation. The 3D network of HM pectin gels is established through junction zones that are stabilized by hydrogen bonding between carboxyl and secondary alcohol groups, as well as hydrophobic interactions involving methyl esters. These gels exhibit thermal reversibility, meaning they can undergo gel-to-sol transitions with changes in temperature. When exposed to hot water, HM pectin gels are soluble, and to prevent lumping, they are often used with a dispersion agent like dextrose [74]. The gelling mechanism of HM pectin is depicted in Figure 2.

The gel formation of high-ester pectins is a complex process affected by various factors beyond just the DE. These pectins typically create gels by linking polymer chains at junction zones, facilitated by hydrogen bonding and hydrophobic interactions among methyl-ester groups. In cases where esters are grouped together, allowing some parts of the molecule to remain as free acids, calcium bridges might also contribute to the gelation process. The factors influencing the gelation process and gel structure of high-ester pectins include the concentration of pectin, its molecular weight, degree of acetylation, branching pattern, pH, ionic strength, water content, type of sugar present, cooling rate, and storage temperature [63]. These parameters play crucial roles in determining the properties and characteristics of the pectin gels [63]. During the gelation process, 3D networks are established, effectively entrapping water and solute molecules within the gel structure. The gel strength of high-ester pectins increases with higher pectin concentration, creating more junction zones and elastic chains [74,82]. The DE determines pH and temperature range for gelling, with higher DE pectins gelling at higher pH and temperature [83]. Acetylation reduces pectin’s gelling ability by hindering interactions [84,85], while neutral sugars can either hinder or enhance gel cohesion through hydrophobic interactions [63,74]. For sugar composition in high-ester pectin, the effect is dependent on the molecular geometry of the sugar interacting with neighboring water molecules [80]. The other factor affecting pectin gelling properties is pH level. Lower pH promotes gel formation by facilitating interactions between pectin molecules [63]. The carboxyl groups on galacturonic acid residues are less dissociated in acidic circumstances, resulting in less electrostatic repulsion between pectin chains. This permits the chains to generate additional hydrogen bonds and form a gel network. On the other hand, an excessively low pH level might cause rapid gelling without sufficient organization, resulting in a weak and poorly organized gel. 

In addition, pectic polysaccharides, such as pectins, are polyelectrolytes whose gelation behavior and interactions with ions are affected by the solution’s ionic strength [86]. Higher ionic strength can change the pH range for gel formation and enhance the creation of stronger gel networks by binding divalent cations such as calcium (Ca^2+^), which function as bridges between pectin molecules [63]. This can also enhance the creation of junction zones between pectin molecules, resulting in stronger gel networks. These cations operate as bridges between the negatively charged carboxyl groups of pectin, further solidifying the gel structure. Meanwhile, lowering the water activity by increasing the concentration of soluble solids to around 65 wt% is preferred, as it speeds up the gelling process and enhances the strength of the resulting gel [87]. When water activity is reduced, gelation occurs faster because there is less water available to hydrate the pectin molecules. As a result, the molecules are forced closer together, resulting in a denser and closer woven gel network. As a result, the final gel strength increases. The cooling rate of gels has been indicated as another factor influencing the pectin gelation rate [63]. Intermediate cooling rates and temperatures are favorable for gel formation as they promote the formation of a network with the highest elasticity. During cooling, hydrogen bonding and hydrophobic interactions between pectin molecules play an important role in gel structure stabilization. A slower cooling rate gives these interactions more time to occur, resulting in a stronger and more elastic gel. Therefore, to explicitly determine the gelling behavior and consequent gel characteristics of high-ester pectin obtained from different sources, the interactions between these parameters and their optimal conditions for desirable gel formation must be investigated. 

### 3.2. Low-Ester Pectin 

Low-ester pectins have historically served as the go-to option for gelling food products in scenarios where high-ester pectins may not be as effective in forming a gel. In addition, low-ester pectins have demonstrated their usefulness in forming stable gels in low-to-moderate sugar and acidic environments. Amidated pectins, which are a subcategory of low-ester pectins, exhibit distinct gelling characteristics, providing a wide array of functional properties in the food industry. These unique characteristics make them versatile choices for various food applications.

**Figure 3:**
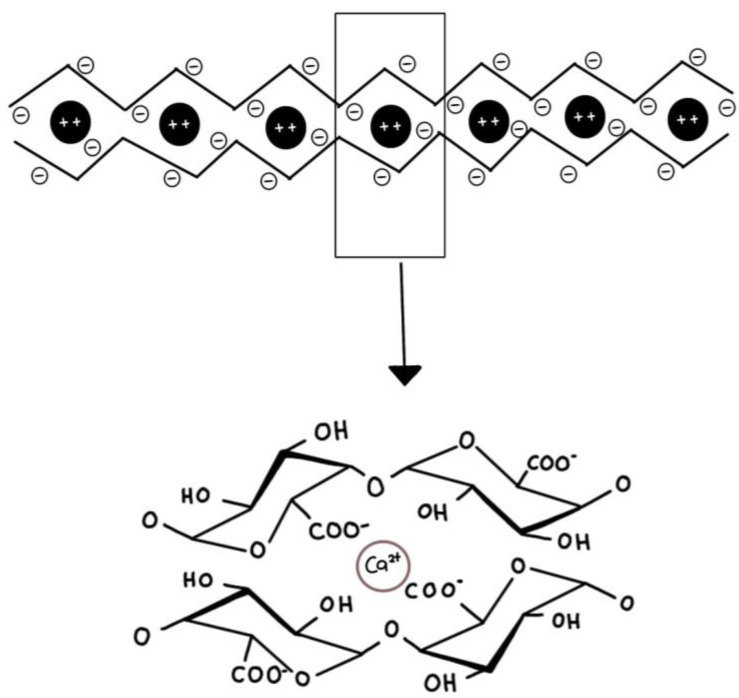
The “egg-box” model illustrating the gelling mechanism of low-methoxyl pectin.

Low-ester pectins with a low degree of methylation (DM) have more free carboxylic acid groups, which interact with Ca^2+^ ions to create a continuous gel network through the “egg-box” paradigm [68]. Figure 3 presents the “egg-box” model, elucidating the gel formation mechanism of low-methoxyl pectin. However, it remains disputed how many contiguous non-methoxylated galacturonic acid residues are necessary for cooperative egg-box formation (6 to 20 residues) [88,89,90,91]. The capacity of the gel to produce stable egg-box junction zones is governed by the presence of extended blocks of non-methoxylated galacturonic acid residues for cooperative Ca^2+^ ion binding [92]. The amount of calcium required for gelation is determined by the DE, size, and distribution of non-methylesterified galacturonic acid, as well as the process parameters [92,93]. Calcium excess can cause pre-gelation or the formation of pectin precipitates.

According to Flutto [63] and Vriesmann [64], the presence of ester, acetyl, or amide groups in pectin disrupts the stabilization of polar groups in the junction zones between neighboring pectic chains, resulting in hindered gel formation. The side chains of pectin may also influence the flexibility of the molecule, preventing aggregation through steric hindrance. Hydrogen bonds and hydrophobic interactions can also impact the ultimate texture of low-ester pectin gels, particularly in conditions of low pH and high soluble solid concentrations. Various parameters influence the gelling of low-ester pectins, including the number and distribution of ester and amide groups, molecular weight, pH, ionic strength, and water activity of the gelling system [63].

Several variables determine the gel strength of low-ester pectins. As calcium bonds can only form in esterification-free areas, lower esterification levels result in greater gel strength. Pectin amidation enhances gelling power, promotes hydrogen bonding, and leads to tougher and thermo-reversible gels with reduced calcium requirements. Amidation increases the gelling ability of low-methoxy pectins by requiring less calcium to gel and making them less likely to precipitate at high calcium levels [94]. The molecular weight of low-ester pectins governs gelation by influencing the number of required connection zones [63]. Pectins with higher molecular weights have more junction zones, leading to faster gelation and reduced syneresis. The length of the pectin chain directly relates to its molecular weight, creating a greater number of junction zones and resulting in stronger gel formation [95,96]. 

The pH of the pectin solution influences gel texture as well as calcium needs. Studies on LM-pectin gel properties [97,98] revealed that lowering the pH < 3 weakened the Ca^2+^ -induced gel for non-amidated pectin but strengthened it for amidated pectin. Interestingly, LM pectin can form gels even at low pH without Ca^2+^ [99]. They proposed that below a certain pH, a conformational transition induces pectin aggregation and gelation when G′ > G″. Additionally, gelation of LM pectin is favored at high pH, as Ca^2+^ bridges require an adequate number of dissociated carboxyl groups [92]. These findings provide valuable insights into the complex gelation behavior of LM-pectin under different pH and Ca^2+^ conditions. Managing calcium requirements is another important parameter influencing gelation, which can be achieved by adjusting the water activity through the addition of sugar or by varying the concentration of soluble solids in the solution [63]. Increasing the solid level reduces the amount of calcium needed while accelerating the gelling process, elevating the setting temperature, and enhancing the final gel strength. However, this approach also leads to a narrower optimal calcium window, prompting practical applications to favor pectin with higher esterification at higher solid levels.

## 4. Types of Pectin Gels

**Figure 4:**
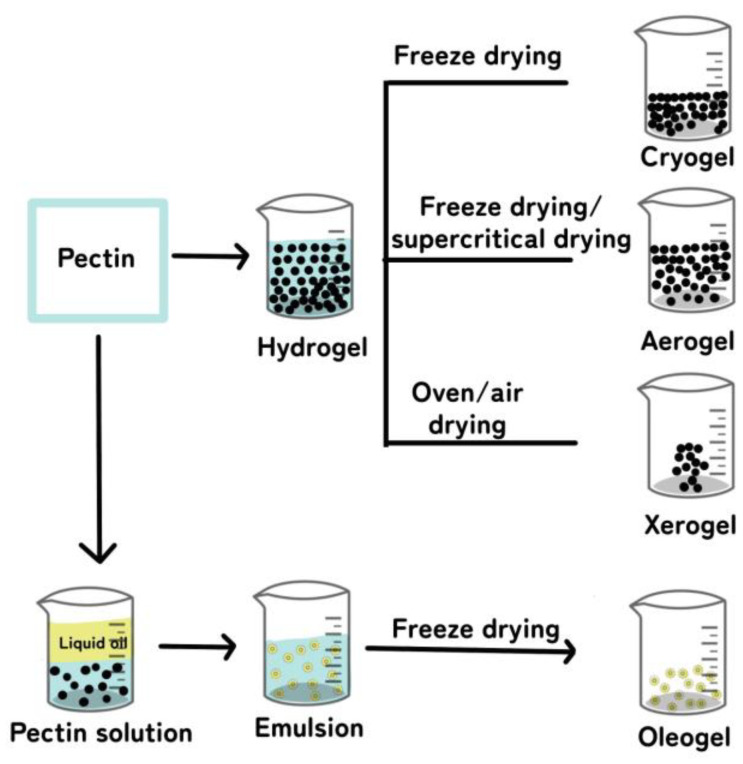
Schematic diagram illustrating various pectin-based gel types according to preparation conditions and varying levels of drying conditions.

Pectin gels exhibit diverse forms, comprising hydrogels, cryogels, aerogels, xerogels, and oleogels, each of which will be explored in detail in the subsequent sections. A visual representation of these distinct pectin gel types is presented in Figure 4, offering a schematic diagram for better understanding.

### 4.1. Hydrogels

Hydrogels are 3D porous materials made from crosslinked hydrophilic polymers, whether natural or synthetic. They possess the ability to absorb substantial quantities of water or biological fluids without dissolution [100,101]. Hydrogels are created by either physically or chemically crosslinking polymer chains, which can be synthetic or naturally derived. The control of hydrogel formation and the enhancement of interactions depend significantly on factors such as pH and charge balance. Most hydrogels, especially those based on polysaccharides, exhibit desirable biocompatibility, biodegradability, tunable structures, and stable physicochemical properties. These unique features of pectin hydrogels enable their wide application in wound healing [102,103], tissue engineering [104], drug delivery [59,105,106], strain sensors [107], supercapacitors [108], aqueous batteries [108], and various other fields. However, hydrogels formed through single chemically or physically cross-linked methods may have poor mechanical properties and weak energy dissipation during deformation. This brittleness limits their potential applications. To enhance their properties, various techniques in preparing hydrogels are utilized, such as ionic gelation, ionotropic gelation, casting, and filtration, offering versatile routes to tailor hydrogels for specific needs, as demonstrated in Table 2.

#### Hydrogel Preparation

Ionic gelation is a commonly employed technique for producing hydrogels, especially those derived from natural polymers such as pectin, alginate, or chitosan. This method is widely used due to its effectiveness and versatility in creating hydrogel networks. This method involves crosslinking polymer chains through ionic interactions with multivalent ions, typically divalent cations such as calcium (Ca^2+^) or zinc (Zn^2+^) [109,130,131]. In this process, the polymer solution is mixed with the crosslinking ion solution, resulting in the creation of a cohesive gel structure. The gelation process occurs when the divalent ions interact with the functional groups (e.g., carboxylate or sulfate groups) on the polymer chains, forming strong ionic bonds. The crosslinking of polymer chains creates a 3D network that traps water molecules, giving rise to the hydrogel structure. The gelation can be triggered by changing pH, temperature, or simply mixing the polymer and ion solutions. In research conducted by Torpol et al. [109], a pectin hydrogel was developed using the ionic gelation method, which involved the combination of chitosan and essential oils with the addition of CaCl_2_. In another study, the ionic gelation method was utilized for microencapsulation-based gelation, enabling the crosslinking of polyelectrolytes (pectin and chitosan) with multivalent ions, including calcium (Ca^2+^), to create the hydrogel bead [132]. The study findings indicated that the hydrogel developed exhibited significant inhibition activity against various harmful bacteria, such as *B. cereus*, *C. perfringens*, *E. coli*, *P. fluorescens*, *L. monocytogenes*, and *S. aureus*. This suggests the potential of the pectin–chitosan hydrogel for antimicrobial applications. 

Ionotropic gelation is a specific type of ionic gelation that relies on the capacity of polyelectrolytes to undergo crosslinking when exposed to counterions, leading to the formation of hydrogels [133]. In this method, the polymer solution is mixed with a solution containing metal cations, such as calcium (Ca^2+^) or aluminum (Al^3+^). The metal cations interact with the carboxylate or sulfate groups on the polymer chains, leading to gel formation [134]. The formation of the hydrogel structure through ionotropic gelation is influenced by factors such as the polymer concentration, crosslinking ions, and ionic strength of the surrounding medium. By adjusting the concentration and types of metal cations used, the crosslinking process can be customized. Ionotropic gelation finds widespread use in various applications, including controlled drug release, encapsulation of bioactive substances, and tissue engineering purposes. In another study by Popov et al. [111], the ionotropic gelation method was employed to create hydrogels using low-methyl apple and hogweed pectin samples with the addition of calcium gluconate. Ionotropic hydrogels are formed when polymers gel in the presence of metal cations. Pectin, with its carboxylate groups, readily forms gels in the presence of metal cations like Ca^2+^. Calcium gluconate, a divalent metal cation, can crosslink with pectin and contribute to its gelling properties. The use of calcium gluconate was preferred over calcium chloride due to its milder taste, improving consumer acceptance. The presence of sucrose was observed to positively influence the creation of pectin gels by stabilizing the crosslinks between pectin and calcium ions. Moreover, sucrose forms hydrogen bonds with water molecules, resulting in the immobilization of free water and promoting the concentration of the polymer environment, thereby facilitating gelation. The study demonstrated that a mixture of both apple and hogweed pectin showed a synergistic effect, contributing to higher gel strength in the hydrogels formed through ionotropic gelation [111].

The casting method involves the preparation of hydrogels by casting a solution or dispersion of hydrogel precursors into a mold or container of the desired shape [135]. Natural polymers, synthetic polymers, or a combination of both can be used as hydrogel precursors. Precursor gelation happens by physical or chemical crosslinking, depending on the formulation. Intermolecular forces like hydrogen bonding and hydrophobic interactions cause gelation in physical hydrogels. Conversely, chemical hydrogels are created through covalent bonding of polymer chains, in which chemical processes generate permanent links between polymer chains. The casting approach provides for exact control over the shape and size of the hydrogel, making it suited for applications such as drug delivery systems, wound dressings, and tissue engineering scaffolds. The casting method of developing hydrogel was demonstrated by Elma et al. [113], who showed good compatibility between CMC and pectin from banana peels that led to stabilization of cross-linking the hydrogel membrane synthesis. The study also showed an increase in the hydrophobicity of the hydrogel membrane due to the addition of banana peel pectin. 

The filtering process includes extruding a mixture of hydrogel precursors through a membrane or filter with precise pore sizes to create hydrogel beads [114]. The extrusion procedure results in the creation of uniformly sized hydrogel beads. Polymers containing crosslinking functional groups, such as alginate or chitosan, can be used as hydrogel precursors [132]. During the extrusion process, the hydrogel precursors interact and create a gel network [136]. The addition of crosslinking agents or ions can improve the gelation process even more. The filtration method is commonly used for the encapsulation of drugs, enzymes, or bioactive compounds, as well as the delivery of therapeutic agents and the immobilization of cells for various biomedical applications. A study by Lee et al. [114] demonstrated the filtration method for preparing hydrogels. The study showed that adding pectic oligosaccharide (POS) resulted in smooth hydrogel beads with fewer surface imperfections. The smoothness was achieved through hydrogen bonding between resistant starch (RS) beads and POS. The study suggests that RS-POS (1.2%) hydrogel beads could be used as an effective carrier for encapsulating *L. bulgaricus* probiotics, offering protection and controlled delivery.

### 4.2. Cryogels

Cryogels are a type of pectin gel formed using a cryotropic gelation process. In this method, pectin solutions are frozen at sub-zero temperatures, and the ice crystals formed act as templates for the gelation process [137]. As the frozen gel is thawed, the ice crystals melt, leaving behind a porous network of interconnected pectin chains [138]. Cryogels have a highly open and porous structure, making them ideal for applications requiring high surface area and rapid mass transfer, such as in food packaging, adsorption, and filtration processes. Cryogels are 3D porous materials formed by a process called cryotropic gelation. The preparation of cryogels involves two main methods: freeze-drying (also known as lyophilization) and film drying, as stated in Table 1. A study by Konovalova et al. [124] demonstrated polymeric cryogels formed through freeze-drying, which involves freezing and thawing the initial solutions to create the gel. In this study, low-methyl-esterified pectin from apples and Heracleum were used as the main components, capable of forming a gel with Ca^2+^ ions. The cryogels are prepared by diffusing pectin into a frozen chitosan solution, resulting in the formation of a pectin/chitosan polyelectrolyte complex. The freeze-drying process shapes the unique macroporous structure of the cryogels, contributing to their special properties and applications. However, the freeze-dying process has been revealed to affect the microstructure and mechanical properties of pectin cryogels [125]. This method creates a cellular, less dense structure with a smooth surface and homogeneous honeycomb-like pores. The freezing temperature influences porosity, with higher temperatures leading to decreased porosity. The process also impacts mechanical properties, reducing density and increasing porosity. Slow freezing produces larger ice crystals, resulting in shorter drying times and lower hardness in the cryogels. Findings by Groult et al. [120] showed that freeze-dried pectin cryogels undergo limited sample shrinkage (10–13 vol%) due to water freezing and ice crystal growth within the sample. However, this creates large pores and a damaged morphology with cracks and macropores. The resulting freeze-dried cryogels have a very low density (0.07 g/cm^3^), high porosity (95%), and high pore volume (13 cm^3^/g). Compared to hydrogel, aerogel, and xerogel, pectin cryogels have the lowest bulk density (0.073 ± 0.003) and volume shrinkage. 

Meanwhile, pectin cryogels formed using the film drying method have been demonstrated in a previous study [126]. The advantage of film drying properties over pectin cryogel properties is that they allow for the modulation of the releasing rate of drugs, such as enrofloxacin in this case. By incorporating high-methoxylated pectin into the cryogel film, the release rate of the antibiotic enrofloxacin was significantly slowed down [126]. Additionally, the two-layer film system, with the top film equilibrated with different NaCl concentrations, further controlled the release rate of enrofloxacin. This demonstrates the potential of film drying to tailor the drug release properties of pectin cryogels, making them suitable for transcutaneous antibiotic delivery applications.

### 4.3. Aerogels

Aerogels are solid structures composed of colloidal or polymeric networks, known for their extremely low weight and exceptionally high porosity, reaching up to 99.9% (*v*/*v*) open spaces [139]. Aerogels are fabricated through a drying process where the liquid within the gel’s pores is substituted with air. These materials can exhibit a wide range of pore sizes, from macro to micro, resulting in high surface areas and low thermal conductivities. Due to their tunable properties, aerogels have garnered attention as versatile nanomaterials. Their unique attributes, such as ultra-low density, high specific surface area, and remarkable acoustic, mechanical, and thermal insulation properties, make them a special class of advanced materials with great potential for bioactive encapsulation and controlled release applications [116]. In addition, bio-aerogel is a remarkable material characterized by its low density, extensive surface area, and porous structure, offering ample opportunities for functionalization. This exceptional feature arises from the abundant hydroxyl groups present on the polymer backbone, enabling straightforward modification and customization of the material’s properties [140]. 

Aerogels have emerged as highly desirable materials for supporting single or multiple component nanoparticles, owing to their adjustable characteristics like pore size, surface area, and density. The narrow distribution of pore sizes and substantial surface areas enable excellent dispersion of nanoparticles. This dispersion leads to enhanced control over the rates of reactant and product diffusion to and from catalytic sites composed of nanoparticles. By combining robust sol–gel chemistry with various preparation methods, such as supercritical deposition, researchers have successfully developed aerogel-supported nanoparticles with exceptional catalytic properties tailored for specific targeted reactions [141]. The drying step is the most crucial stage in the production of aerogels. The majority of research efforts focused on fabricating polymer aerogels have employed supercritical CO_2_ drying and freeze-drying techniques, as evidenced by the data presented in Table 1. It is worth noting that the properties of the final products vary depending on the drying method employed.

Pectin-based aerogels have been extensively studied using supercritical CO_2_ drying. However, this method has some drawbacks compared to freeze-drying, including complexity, expensive raw materials, and high energy and CO_2_ consumption. A study by Méndez et al. [117] investigated pectin hydrogels prepared through a sol–gel process followed by supercritical drying. They analyzed how pectin composition affected the aerogel structure and release properties when impregnated with vanillin. The developed aerogel particles exhibited high specific surface areas and low bulk density. Pectin’s affinity with vanillin influences shrinkage during aerogel formation and the release profile of vanillin, making it a promising carrier for active compounds in food and biomedical applications. Horvat et al. [118] described the synthesis of biodegradable hybrid aerogels using pectin and polylactic acid as wound-dressing materials. These aerogels were loaded with model drugs and oxygen-generating compounds to assess their drug-release properties. Pectin’s high water uptake and swelling ability make it attractive for wound-dressing applications. The addition of polylactic acid improved the material’s stability in simulated body fluid, which is crucial for wound healing. The resulting hybrid material exhibited a highly porous structure with a large surface area, making it advantageous for drug delivery applications. A study by Groult et al. [120] observed that changing the drying method from freeze-drying (cryogels) to supercritical drying (aerogels) creates noticeable structural differences, particularly concerning specific surface area and pore sizes. Supercritical fluids, like CO_2_, exhibit properties between liquids and gases, allowing for a gentle drying process without damaging the network structure. This results in low-density aerogels with high porosity and pore volume, similar to pectin cryogels. However, aerogels have smaller pores, mainly mesopores and small macropores (50–150 nm in diameter), leading to a significantly higher specific surface area (SBET) of 360 m^2^/g, compared to cryogels with a SBET of 10–20 m^2^/g [120].

Recent studies have shown that freeze drying is a cost-effective method for producing polymer aerogels, comparable to supercritical drying. A study by Wu et al. [115] investigated composite citrus pectin combined with cellulose nanofiber to create aerogels for thymol release. During freeze drying, the emulsion structure around oil droplets is destroyed, leaving oil droplet-shaped pores as a template for the aerogel structure. This aerogel maintained thymol activity, reduced susceptibility to oxygen, and provided slow-release properties. The aerogel was tested on fresh edible mushrooms (*Agaricus bisporus*), extending their storage time up to 5 days by adjusting the humidity in the packaging to 97%. In another study, biopolymer aerogel microspheres were fabricated using alginate and pectin crosslinked with divalent cations (Ca^2+^) via the sol–gel method followed by freeze drying [116]. In this study, as the pectin ratio in the aerogels increased, greater porosity and pore size were observed. Moreover, the encapsulated proanthocyanidins within these aerogel microspheres exhibited controlled release behaviors, conforming to both the first-order and Korsmeyer–Peppas models. Notably, aerogels with higher pectin content exhibited stronger antioxidant activity based on radical scavenging and ferric-reducing antioxidant power results.

### 4.4. Xerogels

Xerogels are a specific category of gels that are formed into solid structures by slow drying at room temperature, allowing them to shrink freely during the process [142,143]. Pectin xerogel can be obtained through the removal of the solvent from the hydrogel by evaporation at room temperature or under vacuum conditions. During this process, the solvent is gradually removed, causing the gel structure to collapse, resulting in a solid material with a high content of interconnected pectin chains. Xerogels have a lower water content compared to hydrogels but retain their 3D network structure. They are commonly used in food applications for the encapsulation and controlled release of bioactive compounds and flavors. Xerogels are a type of hydrogel that is prepared by drying the gel at low temperatures to remove the solvent and water, leaving behind a solid porous material. Evaporative drying commonly results in pore collapse due to elevated capillary pressure, leading to materials with high density and low porosity [144]. There are two common methods used to prepare xerogels: oven drying and air drying, as shown in Table 1. 

Oven drying, also known as conventional drying, is another method for preparing xerogels. In this process, the wet hydrogel is placed in an oven at a controlled temperature to facilitate the removal of solvent and water. The controlled environment ensures more precise drying conditions compared to air drying, but it still requires a longer drying time than freeze drying. Oven drying offers a cost-effective approach that can be easily implemented using standard laboratory equipment. The study conducted by Groult et al. [120] investigated evaporative drying in the development of citrus pectin xerogel under vacuum conditions at 60 °C. This process led to a significant shrinkage of over 90% in the volume of the material. Consequently, the drying method created a compact morphology with a high density (~1 g/cm^3^), a relatively low porosity (approximately 30%), and a low pore volume (0.3 cm^3^/g). The pectin xerogels exhibited some degree of porosity when observed through a scanning electron microscope (SEM), but the specific surface area could not be measured due to possible closed pores. Notably, loading efficiency was high in pectin xerogels (94%), indicating that the impregnation time was sufficient to fully load the pectin alcogel. Another study demonstrated the usage of oven drying at 40 °C to produce xerogel consisting of low-methyl pectin and brea gum [129]. The study found the xerogel exhibited a compact and dense structure with good compatibility between pectin and brea gum, and its swelling and erosion behavior were influenced by the external pH, reaching equilibrium states for water absorption and erosion. These properties of the xerogel have implications for its potential applications in medical, food, and industrial uses, given its response to changes in pH and controlled release behavior. 

An alternative method was employed by Mata et al. [127,128], which utilized air drying to remove the solvent from the gels and obtain the desired xerogel structure. These studies developed sugar-beet pectin xerogels, which were later found to be effective in removing heavy metals (cadmium, lead, and copper) from effluents and wastewater in continuous systems. The xerogels show promising potential as a biosorbent for metal recovery due to their high adsorption capacity and stability. In addition, xerogels also exhibit excellent reusability after multiple batch sorption–desorption cycles. The biosorption capacity and mass of the xerogel beads remain largely unchanged even after multiple reuse cycles, making them suitable for metal remediation technologies.

### 4.5. Oleogels 

An oleogel is a type of gel that is formed by structuring liquid oil using a gelling agent. Typically, the gelling agent is a hydrophilic material, such as a polymer or a surfactant, that can interact with the oil molecules to create a 3D network or structure [145]. This network traps and immobilizes the oil, transforming it into a gel-like consistency. Oleogels are often used as fat replacers in various food products to reduce the amount of solid fats like butter or margarine while maintaining desirable texture and sensory properties [146]. They offer potential benefits by reducing saturated fat content and improving the nutritional profile of food products. Pectin oleogels have been developed using two methods: freeze-drying and homogenizing, as demonstrated in Table 1. 

In the freeze-drying method, a stable emulsion of pectin and oil is first formed. The emulsion is then frozen, and the water in it is removed by sublimation, leaving behind a porous structure of pectin and oil. The oil is trapped within the pores, creating an oleogel. This method preserves the original emulsion structure and results in a highly porous and stable oleogel, but it can be time-consuming and requires specialized equipment. A study by Luo et al. [121] investigated the preparation and application of oleogels made with camellia oil, tea polyphenol-palmitate particles, and citrus pectin using the emulsion-templated method. The concentration of citrus pectin had a significant impact on the physical properties of the emulsions, dried products, and oleogels. Higher pectin concentrations led to more stable and viscoelastic emulsions, as well as dried products with a denser structure and increased hardness. The oleogels exhibited enhanced oil binding capacity and gel strength, with a high gel strength (G′ > 17,000 Pa) observed when the citrus pectin concentration exceeded 1.5% (m/v). These polyphenol-rich oleogels also demonstrated strong antioxidant activity. When used as a replacement for butter in cakes, the oleogels achieved a satisfactory overall quality with hedonic scores ranging from 21.49 to 27.58, compared to a score of 32.03 for cakes made with butter. In addition, Pan et al. [123] developed pectin oleogels combined with tea polyphenol ester particles of different fatty acid chain lengths, which were further used in cookie production as a fat replacer. The study found that the fatty acid chain length influenced the characteristics of the oleogels, including appearance, firmness, and gel intensity. When using pectin oleogels as a butter replacement in cookies, the texture and sensory qualities of the cookies changed. At certain replacement levels, cookies made with specific fatty acid chain lengths in the oleogels showed similar qualities to traditional butter cookies, making them a potential alternative for fat replacement in cookies.

In the homogenizing method, pectin and oil are mixed to form an emulsion using a homogenizer. The pectin molecules stabilize the oil droplets within the water phase. The emulsion is then allowed to cool and set to form the oleogel structure. This method is relatively simple and scalable, allowing for controlled manipulation of the gel structure by adjusting homogenization conditions. However, the resulting oleogel may have a lower porosity and specific surface area compared to freeze-dried oleogels. Dong et al. [122] explored the effect of the interaction between ovotransferrin fibrils (OVTFs) and citrus pectin on the properties of oleogel-based pickering emulsions. OVTF–citrus pectin complexes with better stability were obtained at a mass ratio of 3:1 and pH 5.0, exhibiting pearl chain-like structures. Subsequently, oleogel-based OVTF-stabilized pickering emulsions (OEs) and oleogel-based OVTF–CP complex-stabilized pickering emulsions (OCPEs) were developed. In comparison to OE, the combination of OVTFs with citrus pectin in OCPE resulted in greater stability, smaller droplet sizes, a more noticeable gel-like structure, higher viscosity, and superior textural qualities. The OCPE was also employed as a curcumin delivery method, with superior curcumin preservation, a higher rate of lipolysis, and improved bioaccessibility. This novel strategy sheds new insight on how to customize the characteristics of oleogel-based pickering emulsions by leveraging the interaction between protein fibrils and polysaccharides, which might lead to the precise production of emulsions with preferred shapes and properties.

## 5. Crosslinking in Hydrogel

**Figure 5:**
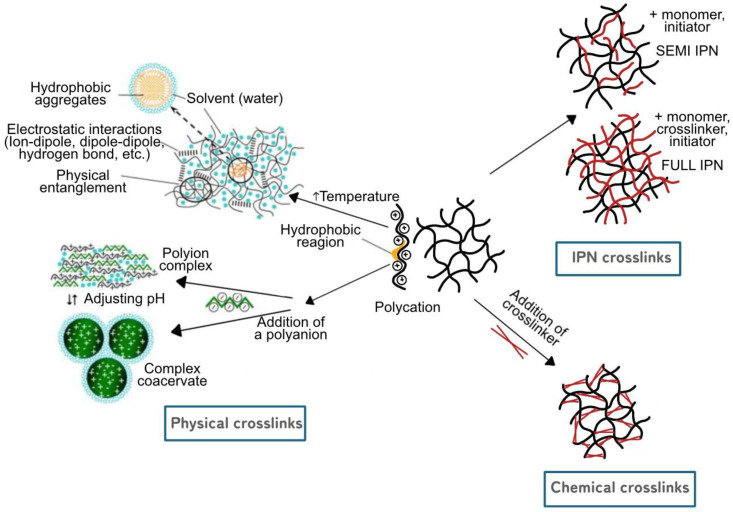
Crosslinking methods, including physical interactions, chemical bonds, and interpenetrating polymer network (IPN) formation, for the preparation of pectin hydrogels. Illustrative figure modified and permitted by [106,147].

The crosslinking of hydrogels encompasses three main processes: physical, chemical, and interpenetrating polymer networks (IPNs), as depicted in Figure 5. Each process imparts distinct characteristics to the hydrogel, making it suitable for specific applications. The choice of crosslinking methods plays a crucial role in determining the hydrogel’s properties, and different crosslinking approaches are employed based on the desired characteristics and applications.

### 5.1. Physical Crosslink Hydrogels

Physical hydrogels are formed through non-covalent interactions, such as electrostatic, hydrogen bonding, and hydrophobic forces, between oppositely charged biopolymers [18,147,148]. These interactions allow for the creation of polyion complexes, where multiple macromolecules come together to form a stable network. The polymer chains in physical hydrogels have strong inter-chain interactions, leading to a cohesive molecular network. At the same time, these hydrogels possess a high affinity for water, encouraging water molecules to access and reside within the gel structure. Due to their reversible and water-sensitive nature, physically crosslinked hydrogels have a short lifespan, typically lasting from a few days to a month when exposed to physiological conditions [18]. This property makes them advantageous for applications where short-term drug release is required, especially in clinical settings, as they do not rely on toxic covalent crosslinking molecules for gelation. In the case of physical crosslinking, the modification process involves interactions that are reversible and do not involve the formation of new covalent bonds. Instead, existing forces such as electrostatic interactions, hydrogen bonding, or hydrophobic interactions are utilized to create the network structure. As an example, a study [149] found that the combination of gelatin and low-methoxyl pectin leads to the formation of a physical co-gel. Electrostatic forces between gelatin and pectin facilitate the interactions, resulting in a reversible physical polyion complex. Gelatin forms the primary network, while pectin is dispersed within. Electrostatic forces facilitate interactions between gelatin and pectin molecules, forming a reversible physical polyion complex with enhanced performance [147]. Furthermore, the study also incorporated glutaraldehyde to achieve 3D crosslinking, which involves the formation of strong and enduring connections through covalent bonds between polymer chains. Upon introduction to the physical polyion complex of gelatin and pectin, glutaraldehyde reacts with specific polymer functional groups, generating new covalent bonds. This process establishes a stable and enduring hydrogel structure characterized by enhanced mechanical strength and water resistance. While physically crosslinked hydrogels may exhibit reduced strength compared to chemically crosslinked ones, they can offer limited stability and durability.

### 5.2. Chemical Crosslink Hydrogels

In contrast to physical hydrogels, chemical hydrogels are formed through the covalent crosslinking of biopolymers at specific sites [147,150]. This crosslinking is achieved using crosslinkers, which act as bridges between polymer chains, resulting in a stable and homogenous network. Unlike physical hydrogels, the synthesis and properties of chemical hydrogels are not solely dependent on pH but can be easily controlled by manipulating the crosslinking process. Chemical crosslinking allows for the modification of various hydrogel properties, including swelling behavior, biodegradability, and mechanical strength. Different approaches, such as the inclusion of small molecules, ionizing radiation, and free radical mechanisms, can be employed for covalent crosslinking [18]. Chemically crosslinked hydrogels offer enhanced stability and durability, making them suitable for longer-term applications. The process of modifying pectin to create its derivatives also encompasses ionic gelation between pectin and another polymer. A study on the combination of pectin and chitosan was investigated by Maciel et al. [151] and Shishir et al. [152]. The study prepared pectin-chitosan hydrogel through the formation of a polyelectrolyte complex. This complex arises from the electrostatic interaction between the negatively charged carboxyl groups (COOH) of pectin and the positively charged amino groups (NH_2_) of chitosan, resulting in the development of a chemically stable hydrogel. In addition, the prepared hydrogel exhibited remarkable moisturizing properties, was biocompatible, and provided a protective effect on skin wounds [110]. Furthermore, an investigation into the synergy of pectin and cellulose integration was undertaken by Chen et al. [153] using an ionic liquid approach, resulting in the development of a chemically crosslinked hydrogel. This research synthesized a natural composite hydrogel by combining flexible pectin and cellulose within an ionic liquid environment. Ionic liquids are often used as solvents to dissolve cellulose due to their ability to disrupt the strong hydrogen bonding network in cellulose [154]. When pectin, which carries negative charges on its carboxyl groups, is combined with cellulose in the ionic liquid, an electrostatic attraction occurs between the positive charges on the cellulose and the negative charges on the pectin. This interaction leads to the formation of a stable composite hydrogel through chemical crosslinking, which involves the creation of ester bonds between cellulose and pectin molecules. This process leads to the development of a hydrogel with a dense network structure and enhanced properties, as described in the study [153].

### 5.3. IPN (Interpreting Polymer Network) Crosslink Hydrogels

IPNs are a unique type of hydrogel that involves the physical entanglement of two or more polymer networks, each with its own distinct properties [147,150]. These networks are interlaced at a molecular scale but not covalently bonded to each other, and they cannot be separated unless chemical bonds are broken. IPNs can be semi-IPNs or full-IPNs, depending on the level of crosslinking between the polymers [18]. In semi-IPNs, one polymer network is crosslinked, while the other is physically associated with the crosslinked network. On the other hand, full-IPNs occur when both polymer networks are crosslinked [104]. IPNs provide a way to combine different polymers, such as natural polysaccharides, proteins, or synthetic hydrophilic polymers, to complement each other’s deficiencies. By utilizing this entangled structure, IPNs offer unique mechanical, swelling, and biocompatible properties, making them valuable in various applications, including drug delivery and tissue engineering. IPNs can be prepared through different routes by combining natural and synthetic polymers, offering versatility and control in hydrogel design. Yan et al. [155] explored the potential use of IPNs consisting of soy protein isolate (SPI) and sugar beet pectin as carriers for probiotic delivery. The researchers employed an enzymatic approach to create the IPN hydrogels and investigated the influence of laccase’s amount as well as the concentrations of SPI and sugar beet pectin on the swelling, textural, and rheological properties of the hydrogels. The authors observed that by altering the laccase quantity and the concentrations of SPI and sugar beet pectin, it was possible to regulate the swelling, texture, and rheological characteristics of the IPN hydrogels [155].

## 6. Potential Application of Pectin Hydrogel in Food Industry

**Figure 6:**
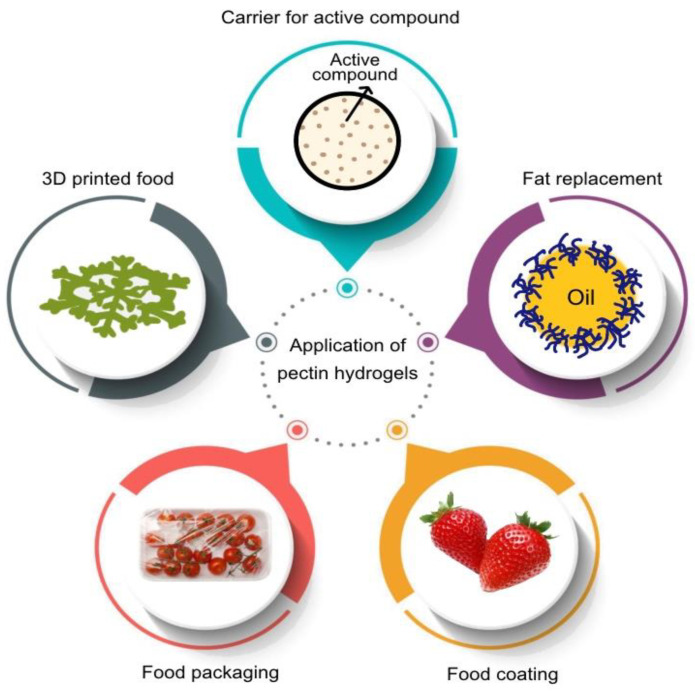
Application of pectin hydrogels in the food industry.

Pectin hydrogels present a wide range of potential applications in the food industry, offering innovative solutions to address various challenges. One of the characteristics of hydrogels is their ability to retain a subsequent amount of water, making them appealing for innovative use in the food industry. Their eco-friendly nature, coupled with inherent biocompatibility, positions pectin hydrogels as an attractive choice for applications focused on minimizing environmental impact and addressing consumer demand for cleaner and healthier food products. The application of pectin hydrogels as agent carriers, fat replacers, 3D-printed food, and food packaging and coating material is depicted in Figure 6 and further explained in this section.

### 6.1. Carrier for Active Compound

One of the primary uses of food hydrogels is the encapsulation of bioactive molecules, including food ingredients, additives, antioxidants, vitamins, probiotics, and drugs. Since hydrogels offer regulated release by virtue of their 3D network, they assure the safety and stability of bioactives during food preparation and storage. Peng et al. [156] achieved the encapsulation of vitamin C in citrus peel pectin hydrogel conjugated with bovine serum albumin. The study observed a 65.31% encapsulation efficiency for vitamin C in pectin hydrogel as a carrier. Another study conducted by Zhou et al. [157] investigated nanohydrogel development involving the combination of pectin with low-density lipoprotein as a carrier for curcumin. The nanogels withstood the challenges posed by stomach acid and various digestive enzymes and facilitated an efficient, controlled release of curcumin over a period of time, enhancing its bioavailability and targeted delivery. In another study, Jung et al. [158] explored the potential of various hydrogel sources derived from low-methoxyl citrus pectins and citrus pectin methylesterase (PME)-modified pectin as carriers for the drug indomethacin. Impressively, the study achieved favorable results in terms of drug encapsulation efficiency, particularly for applications in a drug delivery system targeting the colon through oral administration. Additionally, a novel pH-responsive biopolymer mixture known as Al-P, comprising alginate and pectin, was designed to form a hydrogel at pH levels below 3.0. This innovative approach was demonstrated in the study by Guo and Kaletunç [159]. Notably, the production of disc-shaped particles using this approach was innovative and had the potential to enhance adhesion within the intestines. The hydrogel’s dissolution characteristics adapt to changes in pH within the environment, enabling the controlled and efficient release of bioactive compounds that align with specific physiological conditions. The study aimed to elucidate the factors impacting the dissolution kinetics of Al-P hydrogel and to create mathematical models describing the degradation behavior of these hydrogels under conditions similar to product storage and the lower gastrointestinal tract. Overall, it is evident from the aforementioned studies that pectin hydrogel has the potential to serve as an effective mechanism for delivering active bioingredients into food delivery systems. This property is very effective for increasing the bioavailability of nutrients and functional components, potentially offering consumers health benefits. 

### 6.2. Fat Replacement and Emulsifiers

Pectin hydrogel particles emulate the texture and deformability of fat particles, effectively mimicking the sensory and physical properties of emulsified fats. Notably, pectin-based fat substitutes have emerged, employing various pectin variants with distinct degrees of esterification. For instance, low-methoxyl pectin (LMP), harnessed through calcium gelation, has found application as a fat mimic in products like mayonnaise [160]. On the other hand, high-methoxyl pectin (HMP) played a pivotal role in crafting oil-filled hydrogel granules through controlled phase separation via hydrophobic interactions and hydrogen bonding. This strategic use of HMP serves the purpose of both fat substitutes and emulsifiers [161]. Their flexible and soft nature makes them a healthier alternative to typical fats without compromising taste or texture. Pectin hydrogels can also be used to improve the nutritional profile of meals by increasing the mouthfeel of low-fat products and developing fat-barrier functions. A study by Kavya et al. [162] demonstrated the utilization of pectin sourced from passion fruit rind to produce an emulsion with varying oil content (20–40% oil *v*/*v*). This was carried out to investigate the transformation process from emulsion to emulgel and its consequent impact on the structural and rheological properties. Passion fruit rind pectin demonstrated impressive emulsifying capabilities by significantly lowering the interfacial tension between water and oil. Furthermore, the study highlights passion fruit rind pectin emulgel as a sustainable fat substitute for commercial use, showcasing the potential of pectin hydrogel as a viable fat replacer. In all, the application of pectin hydrogel as a fat replacer and emulsifier is yet to be fully explored and requires more studies for a consistent report.

### 6.3. Three-Dimensionally-Printed Food

Another useful property of pectin is its gelation ability, which allows for the creation of structured meals with certain textures and uses. This feature not only enhances sensory characteristics but also allows for the creation of distinctive meals tailored to consumer preferences. Pectin hydrogels are a significant combination for innovative technologies such as 3D printing. Their structural stability makes them suitable for 3D printing applications in food design, enabling the precise fabrication of complex shapes and customized food products. Among the pectin types, LMP has been proposed by a few studies as a suitable food-ink material for the 3D printing of customizable food simulants. A study by Lu et al. [163] formulated polysaccharide-based hydrogel food inks using ionic crosslinked LMP and cellulose nanocrystalline (CNC). LMP, characterized by its lower degree of methoxylation, typically forms gels through electrostatic interactions with cations like Ca^2+^. The formation of a polymeric network by crosslinking LMP with calcium ions contributed to maintaining the 3D structure of the hydrogels formulated for the food ink in this study. In another investigation conducted by Vancauwenberghe et al. [164], the adjustment of pectin, sugar syrup, and bovine serum albumin (BSA) concentrations was explored to manipulate the desired texture and structural properties of the printed food. The results showed that the viscosity and mechanical properties of the printed food were primarily influenced by pectin and sugar concentrations, while BSA enhanced the gel’s porosity.

### 6.4. Food Packaging

In food packaging, hydrogels are applicable due to their unique properties, such as water retention and controlled release. They prolong the shelf life of perishables, notably fruits and vegetables, by regulating moisture and gas exchange, reducing food waste, and ensuring fresher products. Moreover, hydrogels can also be tailored to release antimicrobial agents or antioxidants, enhancing food preservation and safety. Importantly, they contribute to sustainability by reducing single-use plastics. This section summarizes their various film- and coating-based approaches.

#### 6.4.1. Film-Based Applications

Pectin-based films incorporated with essential oils and plant extracts, such as clove essential oil [165], copaiba oil [166], marjoram [167], and tea polyphenols [168], have been demonstrated to exhibit good antioxidant and antimicrobial activity while also enhancing the film’s water barrier properties, which led to longer preservation of intended food products. In another instance, Torpol et al. [109] successfully encapsulated antimicrobial compounds like garlic and holy basil essential oils in chitosan-pectin hydrogel beads, combating various pathogens. The beads demonstrated the capacity to hinder the growth of *Bacillus cereus*, *Clostridium perfringens*, *Escherichia coli*, *Pseudomonas fluorescens*, *Listeria monocytogenes*, and *Staphylococcus aureus*. Another finding by Nešić et al. [119] demonstrated the promising potential of pectin-TiO_2_ nanocomposite aerogels as an environmentally friendly and effective material for food packaging. These aerogels, prepared through a sol–gel process and supercritical drying, exhibit improved mechanical, thermal, and antimicrobial properties compared to traditional pectin aerogels. Notably, their thermal conductivity is lower than that of air, which is a valuable attribute for temperature-sensitive food storage. The study by Otálora González et al. [169] successfully developed functional composite edible films based on pectin with beetroot and red cabbage powder fillers. These films exhibited favorable physico-chemical, mechanical, and thermal properties and demonstrated color stability over a 30-day storage period, suggesting their potential as smart indicators for edible food packaging applications. Another study by Dudnyk et al. [170] developed a pectin-based sensor incorporating red cabbage as a food-derived material, which represents an innovative and edible solution for food packaging. This sensor operates as a colorimetric indicator of food freshness, demonstrating high sensitivity to gaseous amines. It effectively detects degradation in various food samples, including beef, chicken, shrimp, and fish, with colorimetric changes aligning well with standard degradation markers. The sensor’s ability to correlate visual and measured changes with established freshness indicators like total volatile basic nitrogen and aerobic colony counting highlights its potential as a smart indicator for food packaging, offering both safety and utility.

#### 6.4.2. Coating Applications

Hydrogel coatings have the ability to protect fresh food from deterioration by providing semi-permeable barriers against harmful factors, reducing enzymatic browning and water loss, and can be fortified with minerals, antioxidants, nutrients, vitamins, or probiotics. A study by Muñoz-Labrador et al. [171] investigated the potential use of citrus pectin gels applied as edible coatings for fresh strawberries. The results demonstrated that these pectin gels effectively enhanced the quality of strawberries during storage, reducing moisture loss, changes in acidity, and alterations in color. Furthermore, the utilization of pectin derived from crude cacao shells as a coating for tomatoes demonstrated the capability to postpone quality deterioration, thereby extending the shelf life of the coated samples to 27 days at 4 °C [172]. This underscores the potential of pectin-based coatings to extend the shelf life and preserve the quality of perishable food products like fresh produce and fruits. Additionally, pectin-based coatings enriched with essential oils have been studied to exhibit both antioxidant and antimicrobial effects. These coatings preserve food quality and safety by preventing oxidative degradation and inhibiting microbial growth, offering a natural and eco-friendly alternative to synthetic preservatives. Pectin-based coatings, enriched with essential oils like oregano, rosemary, Mentha piperita, and lemon, have demonstrated efficacy in enhancing the shelf life of various food items, including broccoli, shrimp, and rainbow trout fillets, by mitigating the growth of spoilage microorganisms [173,174,175]. Similarly, research by Nisar et al. [176] also highlighted the remarkable potential of pectin-based coatings enriched with clove essential oil as potent edible coatings for preserving bream fillets during refrigeration. These coatings, with their demonstrated antimicrobial properties, effectively extend the shelf life of the fillets by inhibiting lipid oxidation and suppressing bacterial growth while simultaneously improving the weight loss, water holding capacity, and textural and color attributes of the bream samples. In addition, research also indicates that active compounds can migrate from pectin-based packaging, influencing sensory characteristics. For example, coating carrots with pectin reduced the accumulation of substances such as lignin precursors and flavonoids, which can contribute to undesirable flavors, resulting in improved overall taste and sensory qualities of the carrots [177]. This demonstrates how pectin coatings can positively impact the way food tastes and feels when consumed. 

## 7. Conclusions

This comprehensive review uncovered the distinct gelling mechanisms of pectin, classified into high-ester and low-ester pectins. High-methoxyl pectins form gels through hydrophobic interactions and hydrogen bonding under specific conditions, while low-methoxyl pectins create continuous gel networks through calcium-mediated “egg-box” formations. Both types of pectin hydrogels offer unique properties with vast potential for various food applications. The review has highlighted that pectin’s gelling behavior is influenced by several factors, including degree of esterification (DE), molecular weight, acetylation, pH, ionic strength, and water activity. Understanding these factors and their impact on gel properties is crucial for optimizing the applications of pectin hydrogel in food design. 

Furthermore, the review explored the wide array of pectin gel types, including cryogels, aerogels, xerogels, and oleogels, each offering distinct characteristics with vast potential in diverse fields. Cryogels and aerogels, characterized by their high surface area and porous structures, demonstrate considerable potential for drug delivery and wound dressing applications. Xerogels, with reduced water content while retaining the 3D network, are valuable for encapsulating and releasing bioactive compounds in food applications. On the other hand, oleogels, formed by structuring liquid oil with pectin, serve as fat substitutes in food items, contributing to formulations that are both healthier and nutritionally enhanced. The review also highlighted the significant influence of processing factors, such as ionic interactions, ionotropic gelation, filtration, and drying methods, on the properties of pectin gels. Understanding and optimizing these factors is essential for tailoring gel properties to specific applications and enhancing the efficiency of gel preparation techniques. However, there are still knowledge gaps, particularly in optimizing preparation methods and functionalizing gels with nanoparticles or bioactive compounds. Interdisciplinary collaborations and eco-friendly approaches are recommended to advance the field and unleash the full potential of pectin-based gels in diverse industries, benefiting both consumers and the environment. 

In addition to pectin gel exploration, this paper also discussed the crosslinking mechanisms in hydrogels, including physical, chemical, and interpenetrating polymer networks (IPNs). Physical hydrogels are formed through non-covalent interactions and are suitable for short-term drug release, while chemical hydrogels, formed through covalent crosslinking, offer enhanced stability and control over properties for longer-term applications. IPNs combine different polymer networks to achieve unique properties, but gaps in understanding cooperative gelation mechanisms and the influence of amidation on gel properties remain. Innovative methods, interdisciplinary collaboration, and synergy between different hydrogel preparation techniques offer potential avenues for advancing the field and unlocking new applications in targeted drug delivery, tissue engineering, and food design.

## Figures and Tables

**Table 1:** Sources and extraction methods of pectin.

Source	Pectin Yield (%)	Extraction Methods	References
Lime peel	17.70–26.30	EAE	[28]
Blood orange peel	19.24	MAE	[29]
Sour orange peel	29.10	MAE	[30]
Navel orange peel	15.47–20.44	CE, MAE, UHP	[31]
Apple pomace	3.63–14.50	SW, EAE	[32,33]
Citrus peel	0.15–28.82	CE, SW, UAE	[32,34,35,36]
Grapefruit peel	23.50–27.34	CE, UAE	[37]
Pineapple peel	1.02–2.12	CE, MAE	[38]
Apple peel	3.60–6.40	CE	[39]
Plantain peel	6.20–13.40	CE, EAE	[40]
Pumpkin peel	8.08–10.03	EAE	[41,42]
Mango peel	1.55–21.82	CE, UAE	[43,44]
Watermelon rind peel	11.25–25.79	CE, MAE	[45,46,47,48]
Dragon fruit peel	7.50	MAE	[49]
Cocoa husk	8.00–11.31	CE	[50,51]
Soy hull	26.00–28.00	CE	[52]
Potato pulp	14.34	CE	[53]
Banana peel	15.89–24.08	CE	[54]
Strawberry	4.10–9.00	CE, UAE, EAE	[55]
Redcurrent	2.20–8.80	CE, UAE, EAE	[55]
Blackberry	4.30–9.10	CE, UAE, EAE	[55]
Raspberry	8.70–12.20	CE, UAE, EAE	[55]

**Table 2:** Types of pectin gels and their applications.

Types of Pectin Gel	Composite Material	Methods	Outcomes	Applications	References
Hydrogel	Pectin/chitosan/essential oils	Ionic gelation (Dripping method)	Good antimicrobial activity against six types of microorganism	-	[109]
Hydrogel	Pectin/chitosan	Ionic charge interaction	Good antibacterial and wound healing properties	Tissue regeneration	[110]
Hydrogel	LM apple pectin	-	Low toxicity, improved stability towards elastic and plastic deformation, ability to adhere to macrophages and the non-specific adsorption of blood plasma proteins	Scaffold for tissue engineering	[13]
Hydrogel	LM apple pectin/LM hogweed pectin	Ionotropic	Increased gel strength	-	[111]
Hydrogel	HM apple pectin/Glucono-δ-lactone	-	Great mechanical strength, stronger thermo-reversibility, and higher pH stability		[112]
Hydrogel (membrane layer)	Banana peel pectin/Water hyacinth carboxymethyl cellulose	Casting	Increased hydrophobicity of hydrogel membrane	-	[113]
Hydrogel	LM pectin/Resistant starch/Lactobacillus bulgaricus	Filtration	High storage ability and protective effects on *L. bulgaricus*	Synbiotic encapsulation, protection, and delivery of probiotics	[114]
Aerogel	Citrus pectin/cellulose nanofiber	Freeze drying	Improved tensile and compressive properties	Edible fungus moisture-regulating packaging	[115]
Aerogel	LM pectin/alginate	Freeze drying	Strong antioxidant activity with good controlled released of proanthocyanidins	Matrix for the controlled release of proanthocyanidin compound	[116]
Aerogel	1.Citrus pectin	Supercritical drying with CO_2_	High specific surface and low bulk density	Matrix for the controlled release of vanillin compound	[117]
	2. Watermelon rind pectin				
Aerogel	Citrus pectin/PLA	Supercritical drying	Increased swelling and simulated body fluid (SBF) uptake	Active wound-healing materials	[118]
Aerogel	Pectin/TiO_2_	Supercritical CO_2_ drying	Great mechanical, thermal, and antimicrobial properties	Temperature-sensitive food	[119]
Aerogel	Citrus pectin	Supercritical CO_2_ drying	Low density with high porosity and pore volume resulted in small pores size, mainly mesopores and small macropores	Matrix for the controlled release of theophylline compound	[120]
Oleogel	Citrus pectin/camellia oil/tea polyphenol-palmitate particles	Freeze drying	Improved oil binding capacity and gel strength	-	[121]
Oleogel	Citrus pectin/ ovotransferrin fibrils	Homogenizing	Better stability, smaller droplet size, more prominent gel-like structure, high viscosity, and superior texture properties	Matrix for the controlled release of curcumin compound	[122]
Oleogel	Citrus pectin/tea polyphenol ester	Freeze drying	Increased stability and viscoelasticity of emulsions, improved oil binding capacity and gel strength of the oleogels	Fat replacer in cookies product	[123]
Cryogel	1. Apple pectin/chitosan	Cryotropic gelation (Freeze drying)	Possessed biocompatibility, biodegradability, and low toxicity	Potential medical purposes	[124]
	2. Heracleum pectin/chitosan				
Cryogel	LM pectin/sucrose	Freeze drying	Reduction of ice crystal in gel	-	[125]
Cryogel	Citrus pectin	Freeze drying	High loading efficiency of theophylline compound	Matrix for the controlled release of theophylline compound	[120]
Cryogel	LM, MM and HM pectin/polyvinyl alcohol	Film drying	Ability to keep the enrofloxacin antibiotic inside the matrix and control of the cargo amount in the gel	Can be used for different infectious pathologies and/or treatments	[126]
Xerogel	Citrus pectin	Oven drying	High density, low porosity, low pore volume and compact morphology	Matrix for the controlled release of theophylline compound	[120]
Xerogel	Sugar beet pectin	Air drying	Improved stability and reusability of the gels with good sorption capability of metal compounds	Heavy metal removal	[127]
Xerogel	Sugar beet pectin	Air drying	Good mechanical strength with high continuous biosorption and desorption of copper	Biosorbent for copper removal in a fixed-bed column	[128]
Xerogel	LM pectin/brea gum	Oven drying	Showed good compatibility between both polymers with high gel strength, while also able to respond to the changes in pH of the medium and modify dye release	Matrix for the controlled release of methylene blue dye	[129]

## Data Availability

Not applicable.
